# Sleep disturbances, cognitive decline, and AD biomarkers alterations in early Parkinson's disease

**DOI:** 10.1002/acn3.52089

**Published:** 2024-05-19

**Authors:** Rui Zhong, Caiting Gan, Huimin Sun, Kezhong Zhang

**Affiliations:** ^1^ Department of Neurology The First Affiliated Hospital of Nanjing Medical University Nanjing Jiangsu China

## Abstract

**Objective:**

We aimed to investigate whether each type of sleep disturbances (i.e., pRBD, EDS, and insomnia) is specifically associated with faster decline in global cognition and different cognitive domains among de novo PD patients. We also assessed the influence of sleep disturbances on core AD CSF biomarkers alterations and conversion to dementia.

**Methods:**

Prospectively longitudinal data were obtained from the PPMI cohort. Sleep disturbances and cognition ability were assessed by questionnaires at baseline and follow‐up visits. Generalized linear mixed models were utilized to assess the effect of sleep disturbances on cognitive decline and core AD CSF biomarkers change. The associations between sleep disturbances and conversion to dementia were analyzed using Cox regression analysis.

**Results:**

Baseline pRBD was associated with faster decline in global cognition and all cognitive domains, including verbal episodic memory, visuospatial ability, executive function, language, and processing speed. EDS was associated with faster decline in three cognitive domains, including verbal episodic memory, executive function/working memory, and processing speed. Insomnia was associated with faster decline in global cognition and verbal episodic memory. Meanwhile, pRBD and EDS were associated with longitudinal decrease of CSF Aβ42. Baseline pRBD increased the risk of conversion to dementia. The risk of dementia in PD patients with multiple sleep disturbances also increased compared with those without sleep disturbance.

**Interpretation:**

Sleep disturbances (i.e., pRBD, EDS, and insomnia) were associated with cognitive decline in early PD. EDS and pRBD were associated with decrease of CSF Aβ42. Moreover, pRBD was associated with conversion to dementia.

## Introduction

Although Parkinson's disease (PD) is defined by its motor manifestation, nonmotor symptoms are common and have a profound effect on patient experience and quality of life.^1^ Sleep disturbances and cognitive impairments are among the most common of these symptoms, with major consequences for patients as well as relatives and caregivers.^2^, ^3^ Sleep disturbances affect up to 98% of PD patients.^4^ The sleep disturbances affected in PD include both nocturnal manifestations, such as insomnia, REM Sleep Behavior Disorder (RBD), Obstructive Sleep Apnea (OSA) and Restless Legs Syndrome (RLS), and diurnal symptoms, such as Excessive Daytime Sleepiness (EDS).^5^

Prior literature have reported sleep disturbances, especially RBD, negatively impact cognitive abilities in PD patients,^6^, ^7^, ^8^ whereas others have not.^9^, ^10^, ^11^ Previous cross‐sectional study revealed the relationship between RBD and poor performance in several cognitive domains, particularly in attention and memory in PD patients.^12^ RBD in PD may be an important risk factor for mild cognitive impairment (MCI).^7^ Naismith et al. have noted that EDS is a significant predictor of slowed processing speed in PD patients.^13^ Thus, the type and extent of cognitive deficits seem to vary widely among different types of sleep disturbance in PD. Specific sleep disturbance may have differing effects on different cognitive domains. Most previous studies that investigated the association between sleep disturbances and cognitive abilities were cross‐sectional studies that only included patients in the moderate to severe stages of disease.^12^, ^14^ High‐quality longitudinal cohort studies and evidence from the early stages of PD are relative limited.^14^ Chahine and his colleague reported that p‐RBD could predict greater annual rate of decline in MoCA score during 3‐year follow‐up.^15^ However, data from the same cohort showed that EDS was not longitudinally associated with cognitive dysfunction in early PD.^16^

Besides the PD‐defining synuclein pathology, other age‐related neurodegenerative pathologies can coexist in PD brains including amyloid beta (Aβ) and tau pathology that are classical features of AD.^17^ Increasing evidence showed the diagnostic usefulness of CSF biomarkers in AD, which provides a great impetus to implement CSF biomarkers in other neurodegenerative disorders such as PD.^18^, ^19^ The cerebrospinal fluid (CSF) Aβ 1–42 (Aβ42), total tau (t‐tau), and phosphorylated tau181 (p‐tau) have been reported to be associated with cognitive impairment in PD.^20^ Low levels of CSF Aβ42 of nondemented PD patients predict development of cognitive impairment over time.^21^ CSF AD biomarkers may play a role in the relationship between sleep disturbances and cognitive decline in PD.

On the basis of these considerations, in this study, we aimed to undertake longitudinal assessments in a cohort of de novo PD patients, with a follow‐up period of 5 years. We aimed to investigate whether each type of sleep disturbances (i.e., pRBD, EDS, and insomnia) is specifically associated with faster decline in global cognition and different cognitive domains. We also determined the specific relationship between different sleep disturbances and core AD CSF biomarkers alterations. The influence of sleep disturbances on conversion to dementia over time was also evaluated.

## Methods

### Study participants and study design

The data used in this study were obtained from the Parkinson's Progression Biomarker Initiative (PPMI) database (www.ppmi‐info.org/data).^22^ PPMI is an ongoing international multicenter longitudinal cohort study with aims to identify biomarkers for PD risk, onset, and progression. Details including the aims and methods of the PPMI study have been published previously and are available on the PPMI website.^22^, ^23^ Data were downloaded from the PPMI database in April 2023. In brief, participants include 413 patients with de novo PD and 196 healthy adults. At baseline, PD patients were required to (1) be over 30 years of age, (2) have been recently diagnosed (within 2 y) and be untreated, (3) have at least two of bradykinesia, rigidity, and resting tremor OR have either an asymmetric resting tremor or asymmetric bradykinesia, (4) have dopamine transporter deficit on dopamine transporter imaging, and (5) have Montreal Cognitive Assessment (MoCA) >26 at baseline. PPMI inclusion criteria for healthy adults also require MoCA >26 at baseline.^22^ In addition, all PD patients were followed up for 5 years. The PPMI study and protocols were approved by the local ethics committees, and written informed consent was provided from each participant before being included in the study. All methods in this study were carried out in accordance with relevant guidelines and regulations.

### Clinical and neuropsychological assessment measures

The sleep disturbances assessed include EDS, insomnia, and pRBD. EDS was assessed using the Epworth Sleepiness Scale (ESS), a validated measure of EDS in which participants rate their likelihood of falling asleep in eight situations.^24^ Participants were categorized as having EDS if ESS was ≥10.^25^ Insomnia complaints were evaluated using subitem 1.7 of the Movement Disorders Society‐Unified Parkinson's Disease Rating Scale (MDS‐UPDRS)^26^. Participants were asked: “Over the past week, have you had trouble going to sleep at night or staying asleep throughout the night? Consider how rested you felt after waking up in the morning.” Clinically relevant insomnia was defined as a MDS‐UPDRS subitem 1.7 score ≥2, which was previously used^27^ and shown to have good correlation with other insomnia rating scales.^28^ The presence of pRBD was assessed using the RBD Screening Questionnaire (RBDSQ),^29^ a widely used tool to assess RBD symptoms and has been validated in several populations, demonstrating both high sensitivity and specificity.^30^, ^31^ We defined pRBD as RBDSQ scores >5.^30^

Neuropsychological tests were performed to assess global and domain‐specific cognitive status, including: the MoCA for global cognition; the Hopkins Verbal Learning Test (HVLT) Total Recall, HVLT Delayed Recall, and HVLT Recognition for verbal episodic memory; the Judgment of Line Orientation (JoLO) for visuospatial ability; the letter–number sequencing (LNS) for executive function/working memory; the semantic fluency test for language; and the symbol digit modalities test (SDMT) for processing speed/attention. For all these tests, a higher score implied a better cognitive performance. All these cognitive tests were assessed at baseline and follow‐up visits. Cognitive status of participants was classified based on the criteria used in previous literature^32^, ^33^, ^34^: normal cognition (NC, MoCA >26), MCI (22 ≤ MoCA ≤ 26), and dementia (MoCA <22). PD participants must have had a MOCA score greater than 26 at baseline.

### Measurement of CSF AD biomarkers

Details of sample collection and processing were previously described (http://www.ppmi‐info.org).^35^ CSF Aβ42, total tau (T‐tau), and phosphorylated tau (P‐tau) concentrations were measured through an Elecsys® electrochemiluminescence immunoassay (ECLIA) using a completely automated cobas e 601 analyzer (Roche Diagnostics, Basel, Switzerland).

### Statistical analysis

For the demographic and clinical characteristics at baseline, descriptive statistics were expressed as mean ± standard deviation (SD) for continuous variables and as percentage frequency for categorical variables. For between‐group comparisons of demographic and clinical variables, Mann–Whitney U tests and chi‐square tests were used. Generalized linear mixed models (GLMM) were utilized to assess the effect of baseline sleep disturbances on cognitive performance decline and core AD CSF biomarkers change over time. These models interpreted the correlations between repeated measures and variables over time. The predictive power of sleep disturbances on cognitive decline and core AD biomarkers change was analyzed via interactions with visit time, thus revealing the influence of sleep disorders on cognitive score and core AD biomarkers changes over time. The associations between type or number of sleep disturbances and conversion to dementia during follow‐up were analyzed using Cox regression analysis. The cumulative incidence of dementia among various groups during follow‐up was compared using Kaplan–Meier survival curves. Bonferroni correction was applied for those multiple comparisons between groups. All GLMM and Cox regression analysis were subject to adjustments for confounding variables, including age, gender, education, age of onset, and APOE ɛ4 carrier status. All data were analyzed via SPSS 26.0, and the statistical significance threshold was set at *p* < 0.05.

## Results

A total of 413 patients with de novo PD and 196 healthy adults were recruited at baseline, and 383, 366, 354, 333, and 305 PD patients were followed up at 1, 2, 3, 4, and 5 years, respectively. The baseline demographic and clinical characteristics of the study cohort were described in Table 1. The average age of patient with de novo PD was 61.53 ± 9.68 years, with a male proportion of 65.1%. Patients with de novo PD had a lower score of MoCA (*p* < 0.001), HVLT Total Recall (*p* < 0.001), HVLT delayed recall (*p* < 0.001), HVLT recognition (*p* < 0.001), and SDMT (*p* < 0.001) than healthy adults. There were significant differences between these two groups in the levels of CSF T‐tau (*p* = 0.001), P‐tau (*p* = 0.029), and Aβ42 (*p* < 0.001). Additionally, significantly more PD patients reported pRBD compared with healthy adults (*p* < 0.001).

**Table 1:** Baseline demographic and clinical characteristics of PD patients and healthy adults.

	PD (*n* = 413)	HC (*n* = 196)	*p*‐value
Age (years)	61.53 ± 9.68	60.81 ± 11.22	0.642
Gender, male (%)	269 (65.1)	126 (64.3)	0.838
Education (years)	15.6 ± 2.97	16.03 ± 2.91	0.114
Age of onset (years)	59.66 ± 9.96	—	0.147
APOE ɛ4 carriers (%)	100 (24.2)	50 (25.5)	0.729
MoCA	27.3 ± 2.03	28.23 ± 1.11	**<0.001**
HVLT total recall	45.89 ± 10.64	49.09 ± 10.2	**<0.001**
HVLT delayed recall	45.15 ± 10.82	49.1 ± 10.7	**<0.001**
HVLT recognition	45.19 ± 11.13	48.09 ± 11.2	**0.001**
JoLO	12.09 ± 2.9	12.46 ± 2.79	0.145
LNS	11.57 ± 2.62	11.76 ± 2.74	0.485
Semantic fluency test	50.79 ± 9.93	52.45 ± 10.35	0.135
SDMT	41.46 ± 9.61	46.72 ± 10.54	**<0.001**
CSF Aβ42 (pg/mL)	907.24 ± 397.37	1014.29 ± 498.04	**<0.001**
CSF T‐tau (pg/mL)	166.52 ± 56.9	189.82 ± 78.64	**0.001**
CSF P‐tau (pg/mL)	14.21 ± 5.27	16.83 ± 8.32	**0.029**
Insomnia (%)	95 (23.0)	32 (16.3)	0.058
EDS (%)	64 (15.5)	24 (12.2)	0.286
pRBD (%)	105 (25.4)	25 (12.8)	**<0.001**

Categorical variables are reported as numbers and percentages; continuous variables are reported as means ± standard deviations. *p* values were assessed by Mann–Whitney U tests and chi‐square tests among the groups. The bold emphasis in the table means *p* < 0.05.

APOE, apolipoprotein E; Aβ42, amyloid‐β42; CSF, cerebrospinal fluid; EDS, excessive daytime sleepiness; HVLT, Hopkins Verbal Learning Test; JoLO, Benton Judgment of Line Orientation; LNS, letter–number sequencing; MoCA, Montreal Cognitive Assessment; PD, Parkinson's disease; pRBD, probable REM sleep behavior disorder; P‐tau, phosphorylated tau; SDMT, Symbol Digit Modality Test; T‐tau, total tau.

Insomnia was associated with cognitive decline during follow‐up. The MOCA score in PD patients with insomnia decreased by 0.453 points per annum when compared with those without insomnia (*β* = 0.453, *p* < 0.001) (Table 2). With regard to specific cognitive tests, patients with insomnia experienced greater declines in HVLT delayed recall and HVLT recognition. There is no significant difference in the MoCA score change between PD patients with and without EDS. With regard to specific cognitive tests, patients with EDS experienced significantly greater declines in LNS and SDMT. Additionally, baseline EDS predicted the longitudinal decrease of CSF Aβ42. Baseline pRBD was also associated with cognitive decline over time. Patients with pRBD experienced a reduction in MOCA score of 0.758 points per annum when compared to those without pRBD (*β* = 0.758, p < 0.001). With regard to specific cognitive tests, patients with pRBD experienced significantly greater declines in HVLT total recall, HVLT delayed recall, HVLT recognition, JoLO, LNS, semantic fluency test, and SDMT. Baseline pRBD also predicted the longitudinal decrease of CSF Aβ42.

**Table 2:** Longitudinal association of sleep disturbances with cognitive decline and core AD biomarkers alterations.

Dependent variable	Insomnia		EDS		pRBD	
	*β* [95% CI]	*p*‐value	*β* [95% CI]	*p*‐value	*β* [95% CI]	*p*‐value
MoCA	−0.453 (−0.698 to −0.207)	**<0.001**	−0.14 (−0.427 to 0.147)	0.339	−0.758 (−0.996 to −0.52)	**<0.001**
HVLT total recall	−0.039 (−1.043 to 0.965)	0.939	1.288 (0.119 to 2.457)	**0.031**	−2.072 (−3.042 to −1.101)	**<0.001**
HVLT delayed recall	−1.653 (−2.695 to −0.612)	**0.002**	1.603 (0.389 to 2.817)	**0.01**	−1.335 (−2.346 to −0.324)	**0.01**
HVLT recognition	−1.693 (−2.684 to −0.701)	**0.001**	0.292 (−0.866 to 1.45)	0.621	−1.302 (−2.265 to −0.339)	**0.008**
JoLO	−0.104 (−0.372 to 0.164)	0.447	−0.038 (−0.35 to 0.275)	0.813	−0.963 (−1.221 to −0.706)	**<0.001**
LNS	−0.224 (−0.484 to 0.036)	0.091	−0.745 (−1.047 to −0.443)	**<0.001**	−0.812 (−1.063 to −0.562)	**<0.001**
Semantic fluency test	−0.605 (−1.568 to 0.357)	0.218	−0.887 (−2.009 to 0.234)	0.121	−2.37 (−3.299 to −1.44)	**<0.001**
SDMT	−0.777 (−1.708 to 0.154)	0.102	−2.973 (−4.052 to −1.894)	**<0.001**	−3.664 (−4.557 to −2.772)	**<0.001**
CSF Aβ42	6.85 (−33.435 to 47.135)	0.739	−67.539 (−114.367 to −20.712)	**0.005**	−41.959 (−80.988 to −2.93)	**0.035**
CSF T‐tau	−0.062 (−4.92 to 4.796)	0.98	3.176 (−2.484 to 8.836)	0.271	2.525 (−2.188 to 7.237)	0.294
CSF P‐tau	−0.084 (−0.531 to 0.362)	0.711	0.389 (−0.132 to 0.909)	0.143	−0.011 (−0.444 to 0.423)	0.961

In these models, sleep disturbance is the independent variable, and the cognitive score and CSF biomarker is the dependent variable; age, gender, education, age of onset, and APOE ɛ4 carriers are covariates. The regression coefficients (*β*) and adjusted *p* values were assessed by generalized linear mixed models. The bold emphasis in the table means *p* < 0.05.

Aβ42, amyloid‐β42; CI, confidence interval; CSF, cerebrospinal fluid; EDS, excessive daytime sleepiness; HVLT, Hopkins Verbal Learning Test; JoLO, Benton Judgment of Line Orientation; LNS, letter–number sequencing; MoCA, Montreal Cognitive Assessment; pRBD, probable REM sleep behavior disorder; P‐tau, Phosphorylated tau; SDMT, Symbol Digit Modality Test; T‐tau, total tau.

To determine the potential of sleep disturbances to serve as predictors of conversion to dementia in de novo PD patients, Cox regression analysis was performed. The crude Cox model showed that there is significant difference in the hazard of conversion to dementia between PD patients with and without pRBD (HR 1.327, 95% CI 1.13–3.346, *p* = 0.016) (Table 3). However, no significant predictive effect of insomnia and EDS on the risk of dementia was found. Similar findings were demonstrated by the adjusted Cox models. Patients with pRBD at baseline had a greater risk of dementia compared with those with no pRBD (HR 1.785, 95% CI 1.032–3.087, *p* = 0.038), while adjusting for age, gender, education, age of onset, and APOE ɛ4 carriers. The hazard for dementia in patients with insomnia tended to higher than those without insomnia, but the difference did not reach statistical significance (HR 1.661, 95% CI 0.923 to 2.987, *p* = 0.091).

**Table 3:** Longitudinal association of type of sleep disturbance with conversion to dementia.

	Crude model			Adjusted model		
	HR	95% CI	*p*‐value	HR	95% CI	*p*‐value
Insomnia	1.327	0.741–2.379	0.341	1.661	0.923–2.987	0.091
EDS	1.007	0.491–2.066	0.984	1.067	0.512–2.222	0.862
pRBD	1.327	1.13–3.346	**0.016**	1.785	1.032–3.087	**0.038**

Adjusted models: age, gender, education, age of onset, and APOE ɛ4 carriers as covariates. The HR and adjusted *p* values were assessed by cox regression models. The bold emphasis in the table means *p* < 0.05.

**Figure 1:**
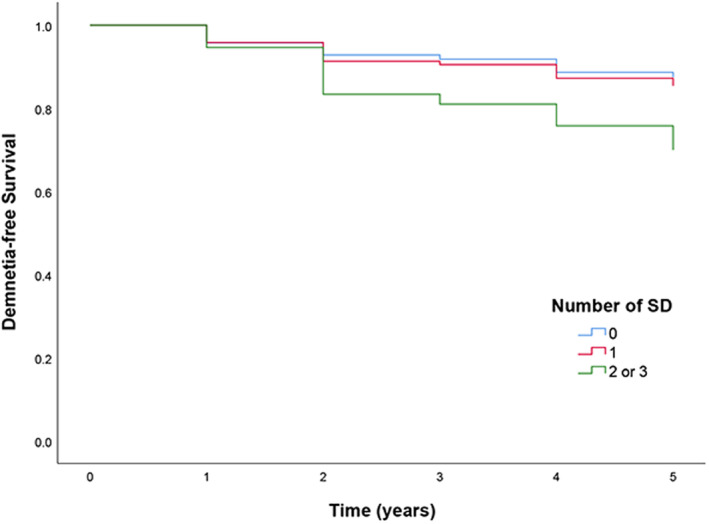
Cumulative probability risk of conversion to dementia in the follow‐up among PD participants. SD, sleep disturbances.

CI, confidence interval; EDS, excessive daytime sleepiness; HR, hazard ratio; pRBD, probable REM sleep behavior disorder;

To determine the association between number of sleep disturbances and risk of conversion to dementia in PD patients, we performed Kaplan–Meier survival curves and Cox regression analysis. PD patients were grouped into whether they reported none, one, and multiple (two or three types) sleep disturbances at baseline. Table 4 shows the incidence of dementia and the HR for conversion to dementia according to the number of sleep disturbance. Figure 1 shows the results of Kaplan–Meier analysis and log‐rank test. The lowest incidence of dementia (11.7%) was seen in patients without sleep disturbance. The highest incidence (23.2%) was seen in patients with multiple sleep disturbances, and the risk of conversion to dementia was 2.47 times higher in these patients than in patients without sleep disturbance (HR 2.47, 95% CI 1.25–4.882, *p* = 0.009).

**Table 4:** Longitudinal association of number of sleep disturbances with conversion to dementia.

Number of sleep disturbance	Dementia incidence (%)	HR	95% CI	*p*‐value
None (*n* = 213)	11.70	Ref		
One (*n* = 144)	13.20	1.018	0.557–1.857	0.955
Multiple (*n* = 56)	23.20	2.47	1.25–4.882	**0.009**

Adjusted models: age, gender, education, age of onset, and APOE ɛ4 carriers as covariates. The HR and adjusted *p* values were assessed by cox regression models. The bold emphasis in the table means *p* < 0.05.

CI, confidence interval; HR, hazard ratio; SD, sleep disturbance.

## Discussion

In the present study, we assessed the longitudinal associations of sleep disturbances with cognitive decline, core AD CSF biomarker alterations, and conversion to dementia among de novo Parkinson's disease patients over time. We found that pRBD in PD was related to faster decline in global cognition and all cognitive domains, including verbal episodic memory, visuospatial ability, executive function/working memory, language, and processing speed/attention. EDS was associated with faster decline in three major cognitive domains, including verbal episodic memory, executive function/working memory, and processing speed/attention. Insomnia was associated with faster decline in global cognition and verbal episodic memory. Meanwhile, pRBD and EDS were observed to be associated with longitudinal decrease of CSF Aβ42. Baseline pRBD increased the risk of conversion to dementia. The risk of dementia in PD patients with multiple sleep disturbances also increased over time compared with those without sleep disturbance.

RBD is characterized by dream enactment and complex motor behaviors during rapid eye movement sleep and rapid eye movement sleep atonia loss (rapid eye movement sleep without atonia) during polysomnography.^36^ The prevalence of RBD ranges from 33% to 46% in PD patients when diagnosed with polysomnography (PSG).^37^ Xu et al. reported a progressive increase in the frequency of RBD in de novo PD patients over time.^38^ Despite growing evidence revealing a cross‐sectional relationship between RBD and cognitive impairment in PD patients, little is known of the specific aspects of this longitudinal association. Folle et al.^39^ reported that pRBD features are a clinical marker for faster cognitive decline in PD patients, but they did not investigated the influence of RBD on specific cognitive domains over time. In this study, we found that RBD was associated with faster decline in global cognitive functioning and all cognitive domains over time. These cognitive profile reflects the effect of RBD on both frontostriatal and posterior cortical deficits. A key question to consider is why patients with RBD are more likely to experience cognitive decline. The mechanisms underlying the association between cognitive decline and RBD in PD remain to be determined. Prior literature suggested that RBD and more severe memory deficits in PD may be epiphenomena of the degeneration of nondopaminergic systems and of altered temporal network.^40^, ^41^ Executive deficits may be due to the abnormalities in subcortical and prefrontal structures in PD.^42^ Relationship between abnormalities in subcortical structures (e.g., caudate nucleus, thalamus, and putamen) and the occurrence of RBD has been established.^43^ Faster decline in visuospatial ability tasks observed in PD patients with RBD could be the consequence of the posterior cortical damage.^41^ We found that pRBD was associated with an increased risk of conversion to dementia in de novo PD patients. Our findings are in line with other longitudinal studies suggesting that the presence of RBD in subjects with PD is an important clinical risk factor for the development of dementia.^44^, ^45^ We also found that PD patients with multiple sleep disturbance had an increased risk of dementia compared with those without sleep disturbances. This finding suggests the multiple sleep disturbances have a cumulative effect on cognitive decline over time in de novo PD patients.

Substantial data are available on the cross‐sectional associations of EDS and insomnia with cognitive impairments in more advanced stages of PD.^46^, ^47^, ^48^, ^49^ Naismish et al. found that EDS was associated with worse performance on processing speed,^13^ whereas another study reported that excessive daytime napping was related with worse executive control performance.^47^ A lack of consensus in the literature on these cross‐sectional associations may be mainly due to methodological differences and limits between the studies. Our study represents the largest of prospective cohort study of de novo PD patients that investigated the influence of insomnia and EDS on global cognition and all cognitive domains over time. We found that EDS and insomnia give rise to faster decline in different cognitive domains. EDS was associated with faster decline in three major cognitive domains, including verbal episodic memory, executive function/working memory, and processing speed/attention. Insomnia was associated with faster decline in global cognition and verbal episodic memory. These results pointed to the fact that EDS is not simply a consequence of insomnia or a poor night's sleep, and the influence of EDS and insomnia on cognitive decline may be driven by a different mechanism. However, using data from PPMI cohort, Amara et al. reported that EDS was not associated with cognitive dysfunction longitudinally.^16^ This may be due to that they only assessed the global cognition with MoCA scores over the 3 years.

One novel aspect of this study is the inclusion of CSF biomarkers data. In the multivariate analysis, the only core AD biomarker that was associated with EDS and pRBD was longitudinal decrease of CSF Aβ42. No association was found with T‐tau and P‐tau. The pathophysiology is multifactorial but aggregation of misfolded α‐synuclein is considered to be a key underpinning mechanism in PD. AD pathology is also found in a considerable portion of patients with Parkinson's disease (PD), particularly those with cognitive impairments and early dementia (PDD).^20^, ^50^ A convergence of evidence suggested that low CSF Aβ42 is predictive of cognitive decline in nondemented PD patients.^21^, ^51^ Conversely, there is limited evidence that CSF levels of tau, either total tau or phosphorylated tau, are a useful predictive biomarker for cognitive decline.^21^ Thus, the longitudinal association between sleep disturbance and cognitive decline may be partly explained by the role of cerebral Aβ deposition in PD.

Strengths of this study include the large sample size and comprehensive longitudinal clinical and biomarker assessments. There are, however, some limitations. While the MDS‐UPDRS subitem 1.7 was previously used to screen clinically relevant insomnia^27^ and shown to have good correlation with other insomnia rating scales,^28^ EDS and RBD were not assessed using any objective measures. As this was a longitudinal cohort study that featured 5 years of follow‐up, some questionnaire scales and biomarker measures were missing due to the long follow‐up time. The inclusion criteria for healthy adults require MoCA >26 at baseline in PPMI cohort; thus, the comparison on cognition between PD patients and healthy adults may be not representative.

## Conclusions

Sleep disturbances (i.e., pRBD, EDS, and insomnia) are associated with cognitive decline in de novo PD patients. EDS and pRBD were associated with longitudinal decrease of CSF Aβ42. Moreover, pRBD was associated with an increased risk of conversion to dementia.

## Funding Information

None.

## Author Contributions

RZ involved in conception and design, statistical analysis, and writing of the manuscript. KZ involved in conception and design, review and critique of statistical analysis, and review and critique of manuscript.

## Conflict of Interest

The authors declare that they have no conflict of interest.

## Informed Consent

Informed consent was obtained from each participant.

## Data Availability

Data used in the preparation of this article were obtained on April 28, 2023, from the Parkinson's Progression Markers Initiative (PPMI) database (www.ppmi‐info.org/access‐data‐specimens/download‐data), RRID:SCR_006431. For up‐to‐date information on the study, visit www.ppmi‐info.org.
